# Prediction of individual differences in non-iridescent structural plumage colour from nanostructural periodicity and regularity

**DOI:** 10.1098/rsos.231804

**Published:** 2024-06-19

**Authors:** Gergely Hegyi, Miklós Laczi, András Wacha, Helga Gyarmathy, Ákos Klein, Balázs Rosivall, Fanni Sarkadi, Gyula Szabó, János Török

**Affiliations:** ^1^ Department of Systematic Zoology and Ecology, ELTE Eötvös Loránd University, Pázmány Péter sétány 1/C, Budapest 1117, Hungary; ^2^ HUN-REN-ELTE-MTM Integrative Ecology Research Group, Pázmány Péter sétány 1/C, Budapest 1117, Hungary; ^3^ The Barn Owl Foundation, Temesvári út 8, Orosztony 8744, Hungary; ^4^ Biological Nanochemistry Research Group, Institute of Materials and Environmental Chemistry, HUN-REN Research Centre for Natural Sciences, Magyar Tudósok körútja 2, Budapest 1117, Hungary; ^5^ Lendület Ecosystem Services Research Group, Institute of Ecology and Botany, HUN-REN Centre for Ecological Research, Alkotmány út 2-4, Vácrátót 2163, Hungary

**Keywords:** coherent scattering, feather structure, phenotypic quality, SAXS, ultraviolet reflectance

## Abstract

Non-iridescent structural plumage reflectance is a sexually selected indicator of individual quality in several bird species. However, the structural basis of individual differences remains unclear. In particular, the dominant periodicity of the quasi-ordered feather barb nanostructure is of key importance in colour generation, but no study has successfully traced back reflectance parameters, and particularly hue, to nanostructural periodicity, although this would be key to deciphering the information content of individual variation. We used matrix small-angle X-ray scattering measurements of intact, stacked feather samples from the blue tit crown to estimate the sex-dependence and individual variation of nanostructure and its effects on light reflectance. Measures of nanostructural periodicity successfully predicted brightness, ultraviolet chroma and also hue, with statistically similar effects in the two sexes. However, we also observed a lack of overall effect of the nanostructural inhomogeneity estimate on reflectance chromaticity, sex-dependent accuracy in hue prediction and strong sex-dependence in position estimation error. We suggest that reflectance attributes are modified by other feather structures in a sex-specific manner, and that within-individual variation in nanostructural parameters exists within or among feathers and this confounds the interpretation of structure–reflectance relationships at the plumage area level.

## 1. Introduction

Sexual selection through mate choice can be an important evolutionary force [1]. Mate choice is typically mediated by conspicuous traits. Although sensory biases can easily explain the evolution of mate choice for many ornamental traits [2], such traits are still generally found to convey information on the body condition of the receiver [3]. Therefore, mechanisms ensuring honest information and condition-dependence represent a central question concerning ornamental traits, a question that has far-reaching evolutionary implications [4,5].

Mechanisms underlying within-species variation in plumage coloration are non-exclusive and often occur in combination [6]. Nevertheless, three rough classes of coloration can be distinguished. The first is pigment-based colour where, even though a structural background is always present [7], the principal source of informative variability may be the metabolism and multi-purpose functioning of the dominant pigment type that differentially absorbs light [8,9]. The second class is individual variation in pigment-free white reflectance. This originates from macrostructural attributes, feather integrity and soiling and its information content includes factors influencing surface size, density, integrity, intactness and cleanness [10,11]. The third class is the chromatic structural colour, which stems from the differential interference of light on ordered nanostructures, combined with the absorption of a basal melanin layer or the diffuse reflectance of the keratin matrix [6]. One category of chromatic structural colour is iridescence whereby the dominant wavelength changes with the angle of viewing relative to illumination. This is typically produced by thin layer structures or photonic crystals [12,13]. Although microstructural and nanostructural attributes have been found to explain individual variation in iridescent colour descriptors [14], there is currently fundamental uncertainty concerning how these colour traits are processed and interpreted by receivers [15].

The second category of chromatic structural colour is non-iridescent. This most often means a reflectance spectrum with a single or double peak in the ultraviolet (UV), blue or green range and the shape of the reflectance curve does not fundamentally change with the angle of illumination. Colour attributes are non-directional because of amorphous keratin nanostructures (globular or spongy) that exhibit short-range order (quasi-order) with a characteristic particle distance so the constructive interference and, therefore, reinforcement of certain, short wavelength ranges is not strongly angle dependent [16]. Prediction of within-species variation of non-iridescent structural colour attributes based on structural measurements is the crucial point to establish the principal proximate basis of signal information content. This has only been attempted in two species so far, in the eastern bluebird *Sialia sialis* [17] and the blue tit *Cyanistes caeruleus* [18]. Both studies processed two-dimensional transmission electron microscopy (TEM) images to infer the structure of the three-dimensional spongy nanostructure (a quasi-ordered network of keratin rods and air channels) that produces colour. One of the studies [17] even applied Fourier transformation to explicitly estimate the periodicity of the spongy structure. The results of the two studies are consistent in that quantity and regularity measures successfully predicted reflectance parameters, but particle size and periodicity measures were unrelated to hue, which is unwelcome news because hue should show a direct causal relationship with these parameters.

The basis of honesty in non-iridescent structural colour could be proposed to be the ability to produce a periodic nanostructure of the appropriate particle distance (the smaller, the more UV) and a sufficient regularity, and, therefore, models of individual quality based on the concept of developmental stability could apply [19]. On the other hand, the spongy nanostructure has been suggested to develop by spontaneous self-assembly, raising questions concerning the limiting factors, information content and condition-dependence of such processes [20]. Furthermore, it is known that feather macrostructural parameters unrelated to the spongy structure (particularly barb density) affect colour saturation and that particle heterogeneity in the spongy structure may alter the perceived hue, thereby confounding the particle distance signal [18]. Therefore, it would be imperative to determine the basis of individual variation and, therefore, information content in this colour type. The failure to estimate hue from sponge periodicity may imply that (i) methods employed so far have been inaccurate or spatially non-representative [21], or that (ii) the periodic nanostructure produces more or less the same colour (by species-specific self-assembly), which is tuned to individually varying hues and saturations by means of other nano- or macrostructural traits, or in some cases, pigments. Under mechanism (ii), attributes of the spongy layer would play minor or negligible roles in colour signalling, laying down an entirely different concept of trait honesty.

When investigating a presumably nanostructural-based colour, representative sampling may represent a key problem. We may measure spongy nanostructure within a cut or narrow section of a single feather barb. However, quantitative and qualitative aspects of the structure may change along the barb. Furthermore, there are possibly dozens of barbs in a single feather and large numbers of feathers in the same plumage area, and these represent levels of variability unexplored by the above approach [22]. However, is there significant variability to explore? Across-season monitoring of colour in the two species subject to the above nanostructural studies revealed significant changes not only in brightness and chromaticity but also in hue, particularly around the reproductive season [23,24]. Although this change may involve seasonally varying thickness and composition of preen gland wax cover [25], it may also indicate the differential abrasion of feather parts, i.e. these studies may indirectly reveal within- and across-feather variation in colour-producing nanostructures.

Several recent studies of non-iridescent structural plumage colour employed small-angle X-ray scattering (SAXS). This technique was developed for the measurement of dominant periodicity and regularity in periodic nanostructures. Importantly, the method can measure the intact feather material without the invasive preparation and two-dimensional slicing that accompanies TEM. In a large multi-species SAXS study, among-species variation in the hue of non-iridescent structural colour has been successfully traced back to the estimated dominant periodicity of the nanostructure, although the relationship is imperfect and in particular, there are UV-blue species in which natural hue is considerably lower than the value predicted from the nanostructure [26]. Nearly all other applications of the technique used small samples and aimed to describe the basic colour-generating mechanism [27–30]. As far as we know, only one study measured a statistically tractable sample of individuals of a single species with SAXS (rump feathers from nestling blue tits). Here, the goal was to record feather structural and colour responses to a brood size manipulation experiment. Neither colour nor SAXS structural parameters showed treatment effects, but the relationships between structural and colour parameters were not assessed [31].

Here, we measure blue tit crown feathers from a sample of adult individuals, males and females, with both SAXS and spectrometry. This is the first attempt to our knowledge to apply this method to the prediction of individual non-iridescent structural colour variation from the periodicity and regularity of the spongy nanostructure. All feather SAXS studies conducted so far were performed using strong and narrow X-ray beams in synchrotrons. This has the advantage to focus on one or a few barbs of a single feather and thereby reduce noise and improve measurement accuracy. However, it raises the issue that within- and across-feather variation in nanostructure remains unknown. This in turn may drastically reduce the ability to predict plumage coloration as the latter always refers to many feathers. Moreover, access to synchrotrons will always be a prohibitive limitation to the spread of this technique in the large-sample world of ecological and evolutionary studies. Here, we use a smaller, laboratory SAXS instrument and a broader X-ray beam. To specifically account for within- and across-sample variation in nanostructure, we make a series of measurements on feather samples (closely arranged feathers) in a matrix design. We successfully trace back all major colour descriptors to nanostructural attributes, including dominant sponge periodicity. It also seems that nanostructural periodicity may play a major role in generating sexual dichromatism.

## 2. Methods

### 2.1. Field methods

**Figure 1.**
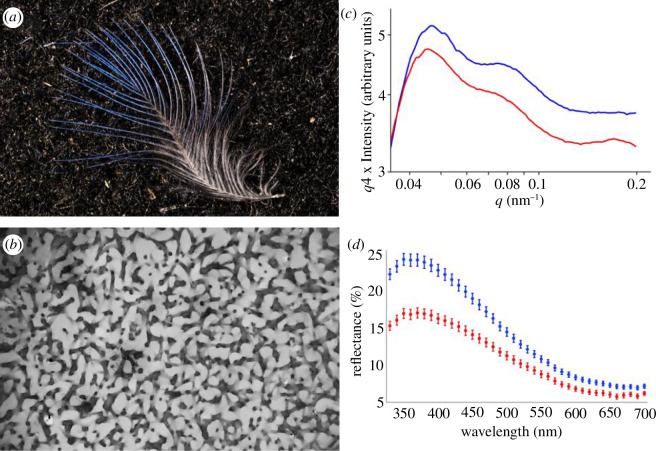
Illustrations of the studied material and the measurement outputs: (*a*) a blue tit crown feather, in which the blue barbs and barb sections containing the spongy nanostructure are towards the tip of the feather; (*b*) TEM cross-section of the spongy keratin nanostructure; (*c*) averaged Porod transformed SAXS scattering curves taken from crown feather samples of males (blue) and females (red); and (*d*) mean reflectance (±s.e.) of males (blue) and females (red) in 10 nm wavelength bins.

We collected crown feather samples in the winters of 2007 and 2022 from blue tits captured in mist nets at feeders baited with sunflower seed. The large time lag between the two capture seasons raises the possibility of environmental and evolutionary changes [32], so we explicitly consider year-related differences in the analyses. The feeders are located within our central nest box plots in the Pilis-Visegrádi Mountains, Hungary. We collected approximately 10 feathers into envelopes. A representative feather is shown in figure 1*a* and a small part of the spongy nanostructure of blue barbs is shown in figure 1*b*. The work was performed under permits from the regional nature conservation authority (KTVF 35464-1/2007, PE-06/KTF/15049-5/2022).

### 2.2. Spectrometry

We did the reflectance measurements using previously validated methods [18,33]. We stacked the feathers atop one another in parallel position on a piece of black velvet, and collected spectral data using a USB2000 spectrometer, a bifurcated fibre-optic probe fitted with a black plastic tube to standardize measurement distance and exclude ambient light, a DH2000 deuterium–tungsten–halogen light source, a WS-1 spectralon white reflectance standard and OOIBase software (Ocean Optics Europe). We took three reflectance measurements in per cent relative to the standard (mean male and female reflectance outputs are shown in figure 1*c*). We then calculated three reflectance variables. Brightness was given as mean reflectance from 320 to 700 nm, UV chroma as the reflectance from 320 to 400 nm divided by brightness, and hue as the wavelength of maximum reflectance. The spectral variables are highly repeatable [18] so they were averaged at the sample level.

### 2.3. Small-angle X-ray scattering

We prepared the samples for SAXS measurements by stacking and fixing the feathers on the sample holder in a parallel position so that the overlapping blue parts would encounter the X-ray beam. Measurements were performed on Creative Research Equipment for DiffractiOn (CREDO) [34], a custom-developed laboratory SAXS instrument. Cu *K*α X-rays (0.154 nm wavelength) were produced by a GeniX^3D^ Cu ULD microfocus X-ray source (Xenocs SA, Sassenage, France), consisting of a 30 W microfocus X-ray tube and a coupled parabolic FOX^3D^ graded Si/W multilayer mirror. In order to reach the required beam size (approx. 0.8 mm) and divergence (<0.03°), an optimized three-pinhole collimation scheme was used [35]. The feather samples mounted on the sample holder platelets were positioned in the evacuated sample chamber of the instrument, and scattering patterns were recorded by a Pilatus-300k CMOS hybrid pixel detector (Dectris Ltd, Baden, Switzerland) placed 2507 mm downstream from the sample. In order to reach the desired high statistical relevance, scattering experiments were carried out in matrix mode, i.e. at 21 different positions of each sample, located on a rectangular grid of 0.8 mm spacing, centred on the 5 mm hole of the sample holder platelets. Exposures of the samples at each such point were performed at least six times, each for 1 min, making in total a minimum of 126 min exposure time for each sample. Before each exposure sequence (consisting of the aforementioned 21 sample positions) dark signal, empty beam, as well as reference samples were measured. Each exposed image was corrected for external and instrumental background, sample self-absorption, detector flatness and measurement time effects. Angle dependence was expressed in terms of momentum transfer defined as


(2.1) q=4πsin(θ)/λ,

where *θ* is half of the scattering angle and *λ* is the X-ray wavelength. Scattering images were azimuthally averaged, yielding scattering curves. After filtering out outliers (affected by artefacts from cosmic radiation or the absence of any feathers), scattering curves belonging to the same sample were normalized on the intensity scale and averaged, yielding a single scattering curve per sample.

The final scattering curves carried similar features as has been found previously in all species with spongy barb nanostructures [26]. Two, relatively broad scattering peaks, attributed to the spongy structures were observed at very small angles (near 0.045 and 0.075 nm^−1^, respectively), on top of a relatively strong, *q* ^−4^ power-law baseline, attributed to larger scattering objects such as melanin pigment particles. The so-called Porod plot (*q* ^4^ times the intensity versus *q*) suppresses this background and enhances the peaks, therefore, we determined their characteristics (position, width and height) by fitting one Gaussian function for the primary and another for the secondary peak in this representation of the scattering curve. Averaged Porod-transformed scattering curves of our male and female samples are shown in figure 1*d*, and the primary and secondary peaks are well visible in these curves.

The parameters of the peaks were determined by fitting the Porod-transformed scattering curve (i.e. *q* ^4^ *I* versus *q*) on the interval 0.038 ≤ *q* ≤ 0.2 nm^−1^ in the least-squares sense with the following mathematical model:


(2.2) $$I(q)\times q^{4}=C+a_{1}e^{\frac{(q-q_{1}{)}^{2}}{h_{1}^{2}}ln\;2}+\alpha a_{1}e^{\frac{(q-\beta q_{1}{)}^{2}}{h_{2}^{2}}ln\;2},$$

where $q_{1}$ and $q_{2}\equiv \beta q_{1}$ are the position of the first and the second peak, $a_{1}$ and $a_{2}\equiv \alpha a_{1}$ are their heights, $h_{1}$ and $h_{2}$ are their half widths at half maximum value, and c is a constant baseline. Fitting was performed in a custom-made Python script using the non-linear Least-squares Minimization and curve-FITting (LMFIT) package [36]. Although the number of scattering units in the illuminated sample volume could not be controlled, the peak height parameters are measured with respect to the constant baseline, thereby the variations owing to the lack of absolute intensity scale are transformed out.

Peak position is inversely related to the periodic repeat distance according to the Bragg law (see §2.4. for details). The width of the peak (measured as half width at half maximum; HWHM) is related to the spatial extent and regularity of the periodic structure in the single spongy matrix. Finally, peak height (amplitude) is related to the strength of coherent scattering. The latter parameter was log(*x* + 10^5^) transformed for both peaks to normalize its distribution.

We fitted scattering curves with two peaks as both primary and secondary peaks have been observed in all species with spongy barb nanostructures [26]. For both primary and secondary scattering peaks, we calculated three parameters. Peak position is inversely related to particle distance according to the Bragg law (see §2.4. for details). For a single spongy matrix, scattering peak width (measured as HWHM) is related to the irregularity of particle diameters and distances. Finally, peak height (amplitude) is related to the strength of coherent scattering. The latter parameter was log(*x* + 10^5^) transformed for both peaks to normalize its distribution.

We checked the repeatability of the six scattering peak parameters by means of re-measurements on 24 male and 24 female samples, performed by completely disassembling the stacked samples and re-mounting them for the second measurement. Therefore, the re-measurement was performed using different ordering, arrangement and partly also different identities of feathers from the same sample. Thereafter, the parameters were standardized for sex (see §3 for sex effects), and correlated between the two measurement rounds. The correlations were moderate to high (*n* = 48, *r* = 0.48–0.86, all *p* < 0.001), indicating a repeatable measurement procedure. The repeatabilities did not differ significantly between male and female samples (sex *x* covariate interactions in general linear models, *F*_1,44_ < 2.83, *p* > 0.100), except for the first-peak position (*F*_1,44_ = 5.74, *p* = 0.021), which was strongly repeatable in both sexes (males *r* = 0.74, females *r* = 0.97, both *p* < 0.001).

### 2.4. Statistical analyses

Statistical analyses were run in Statistica 5.5 (StatSoft, Inc.). We first checked the sex-dependence of all variables using Student’s *t-*tests for mean comparison and *F-*tests for variance comparison, and also checked year-effects using general linear models with year, sex and their interaction as parameters. We sex-standardized the affected variables to a mean of zero and an s.d. of one. We checked relationships between SAXS measures with Pearson correlations. We analysed structural effects on reflectance with general linear models where the reflectance trait was the dependent variable and the structural parameters and their interactions with sex were the parameters.

We checked whether structural parameters explain sexual dichromatism by calculating linear regression residuals for both sexes with the regression parameters of males, the sex with the larger sample size and comparing these residuals between the sexes with *t-*tests. These regressions included the predictors that were significant in the above general linear models. The only change we had to make was that the first-peak position was not significant in the raw regression of male brightness, so we retained the first-peak amplitude for brightness and the first-peak position for UV chroma and hue.

**Figure 2.**
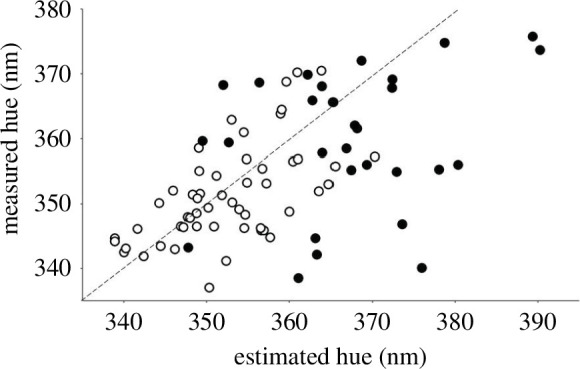
Relationship between the hue values measured by spectrometry and those estimated from SAXS primary peak positions. The dashed line indicates perfect agreement; open circles, males and filled circles, females.

Finally, to predict hue from nanostructural periodicity, we used the following formula:


(2.3) $$\lambda =2\;(2\pi /q)\;n_{avg},$$

where *λ* is the hue, *q* is the position of the first scattering peak, 2π/*q* is the nanostructural periodic repeat distance calculated therefrom using Bragg’s law, and *n*_avg_ is the average refractive index of visible light in the material. Using 1.58 as the refractive index of keratin [37] and 0.57 as the sexually uniform, average density of the spongy layer in our population [18], *n*_avg_ was calculated as 1.33. We then compared the estimated versus measured hue values (plotted in figure 2) in a repeated-measures general linear model. We used data type (estimated versus measured) as a repeated measures factor, hue as the dependent variable, and sex as a factor. We used paired *t-*tests as post hoc analyses.

## 3. Results

### 3.1. Trait correlation structures and confounders

Sex differences were present in all SAXS measures (*t*_80_ > 2.90, *p* < 0.005). For the second-peak position and the first-peak width, the sexes also differed in variance (*F*_1,80_ > 2.05, *p* < 0.025). When running general linear models, year effects were weakly significant (*p* = 0.022) for the first-peak width and non-significant for the other traits (details not shown). Spectral measures showed similar patterns, with strong sex differences in means (*t*_80_ > 4.12, *p* < 0.001), marginal or significant differences in variance (*F*_1,80_ > 1.78, *p* < 0.073) and non-significant or sex-dependent year effects (data not shown). Owing to the variance differences between sexes and the uncertain year effects, we decided to standardize SAXS and reflectance measures for sex (zero mean, unit variance), but not to correct them for the year.

Correlations among sex-standardized SAXS measures were variable (table 1). The strongest relationships appeared between the homologous measures of the first and second scattering peaks, so these certainly cannot be treated as independent. We, therefore restricted the reflectance predictions to first-peak parameters. These three parameters showed medium-sized correlations that were statistically similar between the sexes (*F*_1,78_ < 2.54, *p* > 0.115). When using the SAXS parameters as predictors in a general linear model, collinearity may confound the results. However, running the same models after orthogonalizing the predictors with Varimax-rotated principal components analysis, the significant results we report below remained the same (details not shown here), so collinearity apparently does not affect the conclusions we draw below. The few additional relationships appearing after orthogonalization are mentioned below.

**Table 1.** Pearson correlation matrix of sex-standardized SAXS parameters for the 82 feather samples pooled (53 males and 29 females). (*p < 0.05; **p < 0.01; ***p < 0.001.)

	position 2	width 1	width 2	amplitude 1	amplitude 2
position 1	0.55***	−0.42***	0.19	−0.17	0.09
position 2		−0.22	−0.21	0.31**	0.29**
width 1			−0.63***	0.13	−0.24*
width 2				−0.26**	0.13
amplitude 1					0.83***

### 3.2. Structural predictors of reflectance

**Figure 3.**
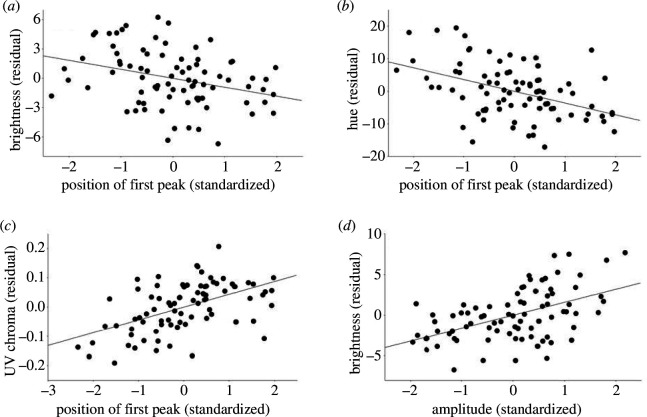
Relationships of reflectance descriptors with SAXS first-peak parameters; first peak position with brightness (*a*), hue (*b*) and UV chroma (*c*) and amplitude with brightness (*d*). Note that the first-peak position is negatively related to particle distance in the spongy layer. Values of SAXS parameters have been standardized for sex, while those of the reflectance variables are residuals from linear models with all significant parameters except for the focal independent variable of the given plot.

The effect of structural predictors (see all results in table 2) did not differ significantly between the sexes for any reflectance trait. The position of the first peak correlated negatively with brightness (figure 3*a*) and hue (figure 3*b*) and positively with UV chroma (figure 3*c*). The first-peak amplitude was positively related to brightness (figure 3*d*) but not related to UV chroma or hue. The first-peak width was not significantly correlated with reflectance. After orthogonalization (details not shown), hue was also predicted by the first-peak amplitude (positive relationship) and the first-peak width (positive relationship).

**Table 2.** Reflectance descriptors in relation to year, sex and SAXS first-peak parameters; general linear models with backward stepwise simplification and parameter reintroduction. (SAXS parameters were standardized for sex before inclusion in the model. d.f., degrees of freedom; *p < 0.05; **p < 0.01; ***p < 0.001.)

	brightness		UV chroma		hue	
	*F*	d.f.	*F*	d.f.	*F*	d.f.
year	2.13	1, 77	45.57***	1, 77	9.04**	1, 77
sex	33.04***	1, 78	69.14***	1, 77	29.36***	1, 77
position 1	9.27**	1, 78	29.23***	1, 77	18.42***	1, 77
width 1	1.63	1, 77	0.03	1, 76	1.45	1, 76
amplitude 1	28.18***	1, 78	0.09	1, 76	2.94	1, 76
year × sex	0.24	1, 76	5.35*	1, 77	6.62*	1, 77
sex × position 1	0.40	1, 77	1.75	1, 76	1.54	1, 76
sex × width 1	0.48	1, 76	0.36	1, 75	0.15	1, 75
sex × amplitude 1	0.97	1, 77	0.69	1, 75	0.36	1, 75

### 3.3. Explanation of sexual dichromatism

**Figure 4.**
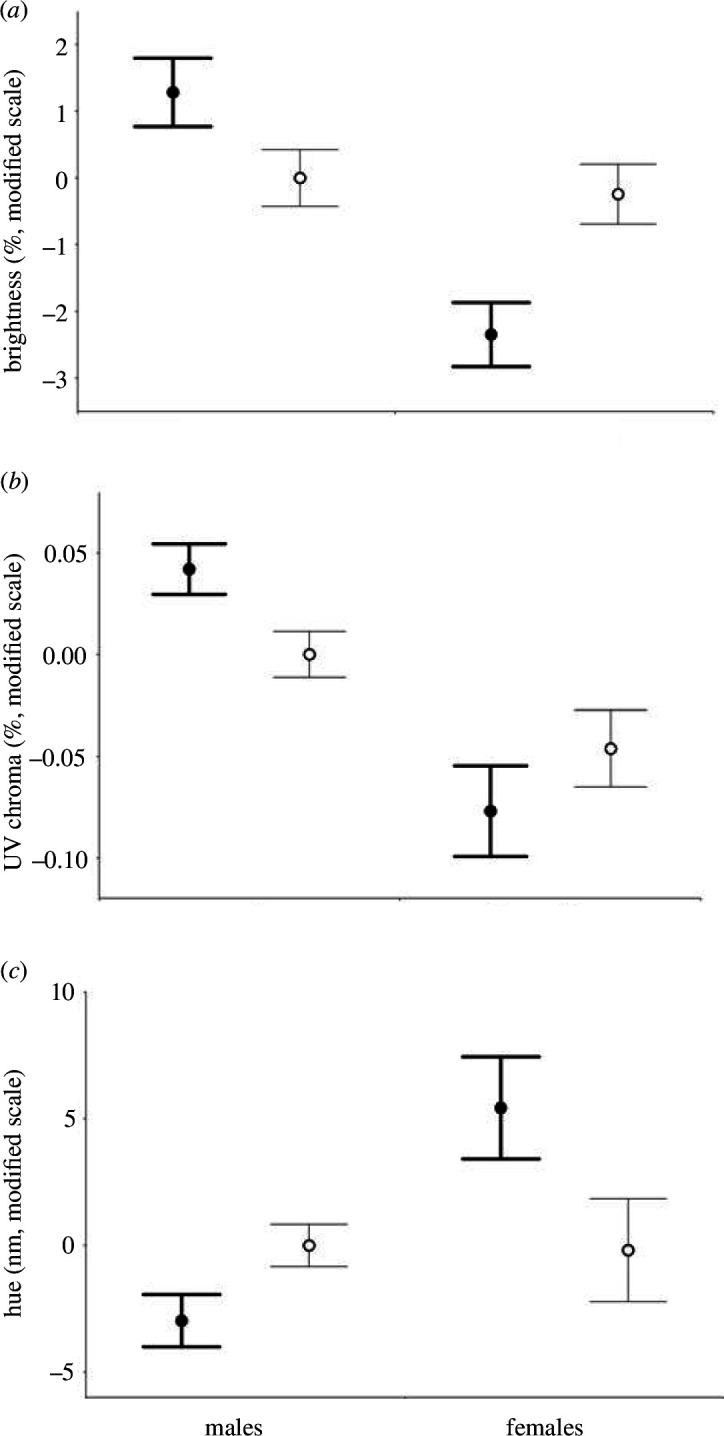
This figure illustrates that nanostructural differences measured by SAXS play important roles in explaining sexual dichromatism in colour attributes, brightness (*a*), UV chroma (*b*) and hue (*c*). Sex differences in reflectance descriptors (mean ± s.e., 53 males and 29 females) are shown before (thick line, filled circle) and after (thin line, open circle) correction for the dominant SAXS predictor using the regression line fitted on male data; means ± s.e. Uncorrected reflectance variables were centred to zero before plotting, while corrected reflectance variables are regression residuals.

There were significant sex differences in both structural and reflectance variables, and these differences were consistent with the direction of within-sex relationships between structure and reflectance. It is therefore valid to ask whether sex dependence in nanostructure explains sex differences in reflectance. After the structural correction of all data with the regression parameters of male birds (see §2), the originally strong sex differences in reflectance variables remained just significant for UV chroma (*t*_80_ = 2.23, *p* = 0.029) and became non-significant for brightness (*t*_80_ = 0.37, *p* = 0.715) and hue (*t*_80_ = 0.10, *p* = 0.921). The residuals and the original data in relation to sex are plotted in figure 4.

### 3.4. Prediction of hue

The estimated versus measured hue values are plotted in figure 2. We found a significant interaction between sex and the repeated measures factor (*F*_1,80_ = 9.02, *p* = 0.004), indicating that the correspondence of estimated and measured values differed between the sexes. Indeed, there was apparently no directional estimation error in males (paired *t*_52_ = 1.34, *p* = 0.186) but a significant overestimation by approximately 8 nm appeared in females (paired *t*_28_ = 3.22, *p* = 0.003). The relationships are positive and do not differ significantly between the sexes (see results for the first-peak position and its interaction with sex in predicting hue in table 2).

## 4. Discussion

### 4.1. Sponge density and brightness

We predicted that spongy layer density would positively correlate with overall mean reflectance (interscatterer distance as an inverse measure of scatterer density was negatively correlated with brightness in our previous electron microscopy study [18]). Indeed, here the relationship between SAXS peak amplitude and spectral brightness was positive in both sexes. Moreover, the two regression lines were practically identical and, therefore, the pooled relationship was similar to the sex-specific ones, suggesting that brightness may carry relevant information to the receivers, for example, on the costly material investment of birds into the nanostructure [38]. The question arises to what extent amplitude reflects the scattering properties of the spongy layer versus the amount of feather material exposed to the beam. Controlling for the Porod background (see §2) is likely to have removed intensity variation owing to incoherent scattering, but we cannot be sure what happens to amplitude if the amount of coherently scattering spongy material in the way of the beam increases. We suggest that this possible confounding role of sample thickness may not be serious for two reasons. First, the number of feathers we put in the sample holder was approximately fixed, being only limited by feather availability in some samples. Second, the diameter of the X-ray beam (approx. 0.8 mm) is one magnitude larger than the diameter of a single barb, the barbs of a single feather are not aligned and not immediately adjacent (see figure 1*a*), and, therefore, the overlapping of individual barbs in a stacked bunch of feathers prepared for SAXS is unpredictable. Therefore, if the number of barbs the ray encountered affected amplitude, we would expect very low repeatability of amplitude between matrix measurements of two independent preparations of the same sample. However, the repeatability was considerable, indicating that amplitude was mainly determined by spongy layer density and not sample density or geometry. We, therefore, feel it justified to conclude that mean reflectance truly increases with spongy layer density. In addition, the first-peak position was negatively related to brightness, that is, birds with less UV-tuned spongy layers were brighter. This is an expected relationship since the scattering and absorption process in the spongy structure and the basal melanin layer exaggerates some wavelengths while removing others [16] and therefore reduces mean reflectance across the visible spectrum.

### 4.2. Sponge regularity and ultraviolet reflectance

UV chroma was positively related to the first-peak position. This is a trivial relationship if peak position is related to hue (see below) as samples with more UV-tuned spongy matrices are expected to show higher relative UV reflectance. We focused our attention on the predictive value of sponge irregularity with respect to chromaticity. The width of the coherent scattering peak is considered to reflect the uncertainty of particle distances and, therefore, the uncertainty of selectively enforced wavelengths. Accordingly, the first scattering peak width could even be considered as a key parameter in our study. This is because a particular mechanism of signal honesty for non-iridescent structural coloration would be the difficulty of producing regular spongy nanostructures and thereby saturated UV reflectance. It would follow that individuals producing more regular structures should display ‘better’ coloration [22]. We, therefore, predicted that peak width, as a measure of nanostrucural irregularity, would correlate positively with hue and negatively with UV chroma, i.e. birds with less regular nanostructures would produce ‘less UV’ coloration. We disappointingly found that the first-peak width showed no significant correlation with reflectance parameters. Although the relationships were in the expected direction (negative for UV chroma and positive for hue), they were weak. After removing collinearity between the predictors, the first-peak width significantly predicted hue (in the expected direction), but not UV chroma. Why did we largely fail to find this expected relationship?

Our peak width estimates (approx. 0.007 for males and 0.008 for females) are consistent with a previous estimate using a synchrotron set-up with much narrower beam (0.008) [26], so measurement imprecision cannot be a serious issue here. Another straightforward explanation could be that UV chroma not only indicates spectral saturation but also hue, owing to the truncated nature of the spectral data used to calculate it. However, if we calculate a unidirectional measure of spectral peak half-width that is relatively immune to this problem, this measure is much less strongly related to hue than UV chroma, but it shows almost the same relationship matrix with SAXS parameters, including no relationship whatsoever with scattering peak width (see the electronic supplementary material for details). Extending this measure to the whole reflectance peak including wavelengths invisible to birds, there is also no robust relationship between full-peak reflectance saturation and scattering peak width (see the electronic supplementary material for details). Therefore, collinearity of hue and UV chroma, or spectral restriction owing to bird vision, does not seem to confound our findings. We cannot control for macro-scale confounders of UV chroma (feather and barb arrangement) when measuring stacked feather samples, so this remains a possible explanation.

We nevertheless suggest that the key to the lack of relationship between HWHM and UV chroma may be within-sample variation in spongy layer parameters. A disturbing finding concerning blue tit crown colour is that strong, nonlinear seasonal changes in parameters, including hue, have been detected [23,39]. This is unexpected because the spongy layer is within the feather barbs and it, therefore, cannot change with abrasion. However, hue could still change with abrasion if there is either (i) systematic, within-feather or among-feather variation in sponge periodicity within the same individual and therefore a gradual shift in periodicity and hue with abrasion, or (ii) random sponge periodicity variation within or across feathers and periodicity-related susceptibility to abrasion owing to different keratin density. In either case, within-individual and between-individual variation in periodicity would jointly contribute to the overall relationship between hue and periodicity and, therefore, also between reflectance saturation and periodicity variation. However, does this affect the direction of the overall relationship? It does, if larger scale variability in sponge periodicity within the sample increases the reflectance peak width and, therefore, chromaticity, as predicted by some theoretical models [40,41], while local irregularity of sponge periodicity in any point of the sample reduces chromaticity as per our original prediction. Given the potentially opposite direction of peak width–UV chroma relationships on different scales, the lack of sample-level relationship is not surprising. Analyses of the estimation error of the first-peak position (a measure of within-sample sponge variation; see the electronic supplementary material for details) support the notion that sex-dependent within-sample variation in sponge periodicity exists that may modify or swamp the relationships between SAXS peak width and reflectance variation. Further work is needed to clarify the levels of nanostructural variation within and across feathers in both sexes, and the role these levels play in determining the saturation of coloration. Only after this work will it be possible to draw conclusions concerning the information content of colour saturation. Another question is how receivers process the plumage reflectance of a single individual if it varies among different UV-blue plumage areas and also within areas, as shown by our results. Do they integrate colour expression into a single value or do they also consider inhomogeneity and potentially ‘outlying’ local elements [42]?

### 4.3. Sponge periodicity and hue

The spongy or globular nanostructures in the barbs of feathers with non-iridescent structural colour are chromatic because the periodic nanostructure reinforces certain wavelengths, most frequently in the UV range. Indeed, in systems with no other components but the spongy structure, the selectively reinforced wavelength can be mathematically predicted from the dominant periodicity of the spongy structure [27]. It may logically follow that the dominant wavelength reflected by the tissue may correlate with the particle distance of the spongy layer, and there are supportive results at the species level for both sphere- and channel-type nanostructures [26]. Therefore, we strongly predicted that particle distance in the spongy layer would positively correlate with the wavelength of the reflectance peak (hue). We also predicted that differences in particle distance would predict differences in hue between males and females.

The results are superficially supportive of the predictions. We found that the first-peak position (a measure reciprocally related to the dominant particle distance) was negatively related to hue, with no significant sex differences in this relationship. In addition, predicted hue was of similar magnitude as the measured hue and the two were positively correlated. However, the accuracy of prediction was significantly worse in females than in males. A sex-dependent estimation problem is also suggested by the pattern that (in contrast to brightness and UV chroma) correcting hue for macrostructural periodicity in females using the regression line of males not only removed the sexual dichromatism but resulted in non-significantly reversed dichromatism. The most parsimonious explanation to the sex-dependent hue estimation accuracy is that in males the observable hue of the crown is determined mainly by the spongy layer of barbs, while in females, there is considerable role for other structural elements. Indeed, based on TEM images of a sample of individuals [18], crown feather barbs contain considerably less spongy material in females than in males (Hegyi G., Laczi M., Kötél D., Csizmadia T., Lőw P., Rosivall B., Szöllősi E., Török J. (2024) unpublished data). Furthermore, the ratio of blue and non-blue feather parts is much smaller in females than in males, and blue part length contributes to the explanation of sex differences in hue [18]. However, there may also be a greater degree of within-sample variation in sponge periodicity in females (see previous section) and this may certainly make hue prediction more difficult in this sex.

## 5. Conclusion

According to the raw statistical results, SAXS measures of nanostructural periodicity successfully explained both individual and sex differences in the spectrometric measures of the blue tit crown. However, we need to be extra careful when extrapolating from these results for at least three reasons. First, the variability of two of the three SAXS measures we used (first-peak position and first-peak width) was drastically greater in females than in males. Whereas UV chroma and hue followed this direction of variability, brightness showed a reversed pattern, with greater variability in males than in females. Second, the accuracy of hue prediction was drastically lower in females than in males. Third, a measure of uncertainty in predicting sponge periodicity showed drastic sex differences in mean and variability, and it correlated weakly negatively with our measure of local sponge irregularity.

We can certainly conclude that nanostructural periodicity plays an important role in determining individual differences in UV-blue structural plumage coloration, including hue. The periodic, quasi-ordered spongy layer is apparently characterized by low material and energetic costs ([22; but see [37]) and seamless self-assembly [20], so it remains difficult to explain how coloration predicted by this trait shows condition-dependence [43,44], hormonal-dependence [45] and even viability-indicator value [46]. However, the expression ‘this trait’ remains uncertain because we have difficulties explaining several correlations among our SAXS measures. Some of our results even suggest that both nanostructural and spectral measures taken from a feather sample reflect a combination of local nanostructural characteristics and within-sample nanostructural variation. Furthermore, sex differences in hue prediction accuracy may also indicate the role of other structural elements besides aspects of the spongy nanostructure [18].

Therefore, as suggested previously [18,47], non-iridescent structural feather reflectance may be similar to other coloration types in being determined by multiple different feather attributes acting on different levels [7,48,49]. In addition, the role of structural levels may differ between sexes and cause sex-dependent information content. This means that the task is not only to structurally explain sexual dichromatism [18,50] but also to consider the possibility of sex-dependent structural mechanisms. Indeed, blue tits have been shown to have similar mate preferences for [51], but different parental care responses to, male versus female crown reflectance [52]. This seems logical as mate choice and parental care belong to different phases of the yearly colour degradation cycle [23] and may, therefore, correspond to different colour information content, but the sex-dependence of the relationships may also stem from inherent differences in information content without any abrasion. Future studies of UV-blue plumage coloration should, therefore, focus on the relative roles of within-feather, among-feather and among-individual variation in colour determination, on the condition-dependence and quality-indication value of the different structural pathways and variation levels [31], and the proximate background of the drastic sex-dependent component correlation structures we described here.

## Data Availability

Data are available as the electronic supplementary material [53].
